# Radical SAM Enzymes in the Biosynthesis of Ribosomally Synthesized and Post-translationally Modified Peptides (RiPPs)

**DOI:** 10.3389/fchem.2017.00087

**Published:** 2017-11-08

**Authors:** Alhosna Benjdia, Clémence Balty, Olivier Berteau

**Affiliations:** Micalis Institute, ChemSyBio, INRA, AgroParisTech, Université Paris-Saclay, Jouy-en-Josas, France

**Keywords:** RiPPs, enzyme mechanism, natural products, iron-sulfur proteins, ribosomally synthesized and post-translationally modified peptides, radical AdoMet, iron sulfur, radical SAM

## Abstract

Ribosomally-synthesized and post-translationally modified peptides (RiPPs) are a large and diverse family of natural products. They possess interesting biological properties such as antibiotic or anticancer activities, making them attractive for therapeutic applications. In contrast to polyketides and non-ribosomal peptides, RiPPs derive from ribosomal peptides and are post-translationally modified by diverse enzyme families. Among them, the emerging superfamily of radical SAM enzymes has been shown to play a major role. These enzymes catalyze the formation of a wide range of post-translational modifications some of them having no counterparts in living systems or synthetic chemistry. The investigation of radical SAM enzymes has not only illuminated unprecedented strategies used by living systems to tailor peptides into complex natural products but has also allowed to uncover novel RiPP families. In this review, we summarize the current knowledge on radical SAM enzymes catalyzing RiPP post-translational modifications and discuss their mechanisms and growing importance notably in the context of the human microbiota.

## Introduction

Canonical radical SAM enzymes possess a radical SAM domain defined by the Pfam identifier: PF04055. Currently, according to the EFI (enzymefunction.org) and SFLD (http://sfld.rbvi.ucsf.edu) databases, there are more than 220,000 radical SAM enzymes predicted to be involved in 85 types of biochemical transformations. However, we still have a limited understanding of their mechanisms and the reactions they catalyze.

**Figure 1:**
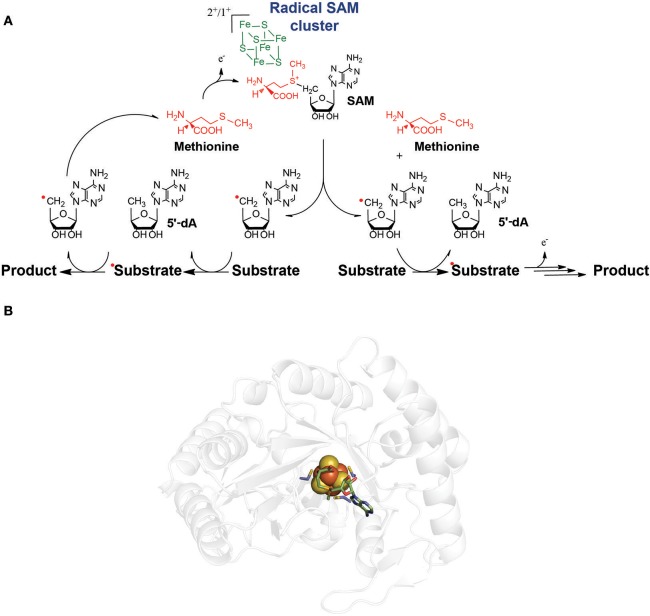
**(A)** General mechanism of radical SAM enzymes. The radical SAM [4Fe-4S] clusters interacts with *S*-adenosyl-L-methionine (SAM). After the reductive SAM cleavage, the 5′-dA radical species is formed and generally abstracts a substrate H-atom. A radical substrate intermediate is formed which, after subsequent rearrangements, lead to the product. In most of the cases, SAM is used as a co-substrate (right pathway). However, several enzymes recycle SAM during catalysis (left pathway). **(B)** Structure of a radical SAM enzyme (Spore photoproduct lyase, PDB:4FHD, Benjdia et al., 2012) showing the radical SAM [4Fe-4S] center (in orange and yellow spheres) in interaction with the SAM cofactor.

The founding members of this enzyme family share as common features: a conserved motif composed of three cysteine residues defined as: CxxxCxxC (where x denotes any amino acid residue), the requirement of a redox active [4Fe-4S] cluster and of *S*-adenosyl-L-methionine (SAM) (Broderick et al., 2014; Benjdia and Berteau, 2016) (Figure 1). However, considerable variations in this cysteine motif have been reported in the last years (Berteau and Benjdia, 2017). Similarly, while the first radical SAM enzymes characterized such as lysine amino mutase (LAM) (Frey et al., 2008), pyruvate formate lyase activating enzyme (PFL-AE) (Knappe and Schmitt, 1976), ribonucleotide reductase activating enzyme (RNR-AE) (Eliasson et al., 1990) and spore photoproduct lyase (SPL) (Chandor et al., 2006; Chandor-Proust et al., 2008; Benjdia et al., 2012, 2014; Berteau and Benjdia, 2017) contained a single [4Fe-4S] cluster, a growing number of radical SAM enzymes have been reported to possess additional iron-sulfur clusters such as biotin synthase (Cosper et al., 2002, 2004), lipoate synthase (Cicchillo and Booker, 2005) or anaerobic sulfatase-maturating enzyme (Benjdia et al., 2007b, 2008). More recently, novel radical SAM enzymes have been shown to contain, in addition to the radical SAM domain, a cobalamin-binding domain, further expanding the structural and chemical versatility of radical SAM enzymes (Pierre et al., 2012; Benjdia et al., 2015; Marous et al., 2015; Ding et al., 2016b; Parent et al., 2016).

Initially, radical SAM enzymes have been associated with the anaerobic life-style. Indeed, most of the founding members of this enzyme family are involved in bacterial anaerobic metabolism (e.g., PFL-AE Knappe and Schmitt, 1976, RNR-AE Knappe and Schmitt, 1976, HemN Layer et al., 2002). In addition, in several instances, radical SAM enzymes support oxygen-independent alternatives to aerobic pathways such as the activation of sulfatases (Benjdia and Berteau, 2016) or the biosynthesis of thiamin (Jurgenson et al., 2009; Challand et al., 2010). Finally, these enzymes are *in vitro* oxygen sensitive because of the presence of a [4Fe-4S] cluster coordinated by only three cysteine residues and the radical chemistry they catalyze. However, genomic and metagenomic studies have revealed their broad distribution from bacteria to human, where they play essential physiological functions (Wei et al., 2011; Glatt et al., 2016).

**Figure 2:**
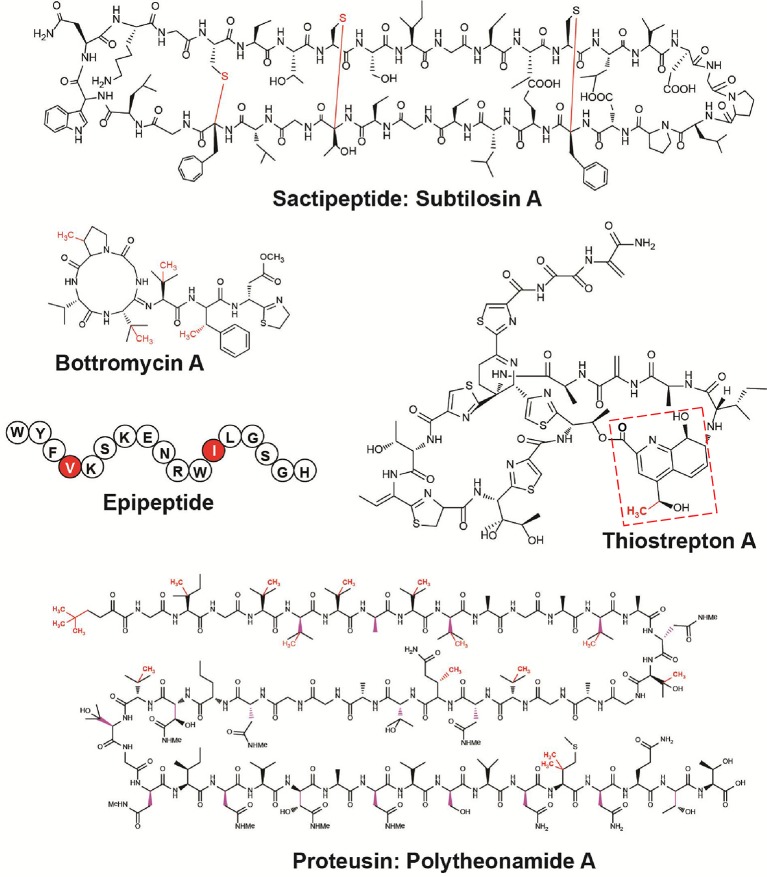
Structures of RiPPs post-translationally modified by radical SAM enzymes. In red are highlighted the modifications catalyzed by radical SAM enzymes. Subtilosin A (thioether bonds), bottromycin A (methylations), thiostrepton A (methylation) (the quinaldic acid moiety is indicated by red dashed lines), epipeptide (epimerization), and polytheonamide A (epimerization and methylations).

Remarkably, during the last 5 years, with the emergence of the RiPP family, the biosynthetic importance of radical SAM enzymes has been more and more recognized (Arnison et al., 2013; Hetrick and van der Donk, 2017). Delineated in 2013, the RiPP family currently encompasses more than 20 classes of natural products with prominent examples such as lantipeptides, cyanobactins, and microcins (Arnison et al., 2013). Interestingly, several classes of RiPPs including thiopeptides (Kelly et al., 2009; Zhang et al., 2011a; Pierre et al., 2012; Ding et al., 2017a), bottromycins (Huo et al., 2012), sactipeptides (Zheng et al., 2000), proteusins (Freeman et al., 2012; Morinaka et al., 2014), and epipeptides (Benjdia et al., 2017b) rely on radical SAM enzymes for their biosynthesis (Figure 2). The radical SAM enzymes involved in these different biosynthetic pathways catalyze a surprisingly wide range of post-translational modifications including methylation, thioether and carbon-carbon bond formation, complex rearrangements and epimerization. This review focuses on the recent mechanistic insights gained on radical SAM enzymes catalyzing RiPP post-translational modifications and highlights their growing importance, notably in the context of the human microbiota.

## Radical SAM enzymes involved in RiPP methyl transfer reactions

### B_12_-dependent radical SAM enzymes: TsrM & PoyC

In RiPP biosynthetic pathways, several radical SAM enzymes have been identified as catalyzing unusual methyl transfer reactions. Interestingly, these enzymes, involved in the biosynthesis of thiostrepton A (Kelly et al., 2009; Pierre et al., 2012; Benjdia et al., 2015), polytheonamide A (Freeman et al., 2012) and bottromycin A (Huo et al., 2012) (Figure 2), have been predicted to possess a cobalamin-binding domain. This cobalamin-binding domain was presumed to coordinate methyl-cobalamin (also called vitamin B_12_) and to be responsible for the transfer of a methyl group to the respective substrates of these enzymes. However, the first experimental proofs supporting these hypotheses have been reported only recently (Werner et al., 2011; Pierre et al., 2012). The first enzyme studied, TsrM, has been identified in the complex biosynthetic pathway of thiostrepton A (Kelly et al., 2009), an antibiotic and anti-cancer agent isolated from *Streptomyces laurentii* more than 60 years ago (Donovick et al., 1955). Its biosynthetic gene cluster revealed a complex pathway involving 20 genes in addition to *tsrA*, the gene encoding the peptide precursor (Kelly et al., 2009). Thiostrepton A is unique among thiopeptides (polythiazolyl antibiotics) by containing a quinaldic acid moieties appended to its core structure making a bimacrocyclic structure (Figure 2). Pioneer studies by Floss and collaborators pointed that the quinaldic acid moiety originates from tryptophan and that the first biosynthetic step was likely the formation of 2-methyl tryptophan (2-Me-Trp) (Zhou et al., 1989). Of particular note, it was established by feeding experiments that the methyl group was transferred from methionine with a net retention of its configuration (Zhou et al., 1989), suggesting a transient methylation of the enzyme during the synthesis of 2-Me-Trp.

Among the 20 genes potentially involved in thiostrepton biosynthesis at least five: *tsrM, tsrN, tsrQ, tsrS* and *tsrV* (also called *tsrT, tsrU, tsrE, tsrD*, and *tsrA* Liao et al., 2009) are predicted to be required for the formation of the quinaldic acid moiety (Duan et al., 2012). Biochemical characterization of TsrN (Duan et al., 2012) and TsrV (Kelly et al., 2009) confirmed their involvement in the biosynthesis of the quinaldic acid moiety and showed them to be an oxidoreductase and an aminotransferase, respectively. Two putative methyltransferases, TsrP and TsrM were identified as potential candidate for the synthesis of 2-Me-Trp. However, since TsrM has a radical SAM domain, it was proposed to be the most likely candidate to catalyze the unusual C2 methyl transfer reaction on tryptophan (Kelly et al., 2009).

**Figure 3:**
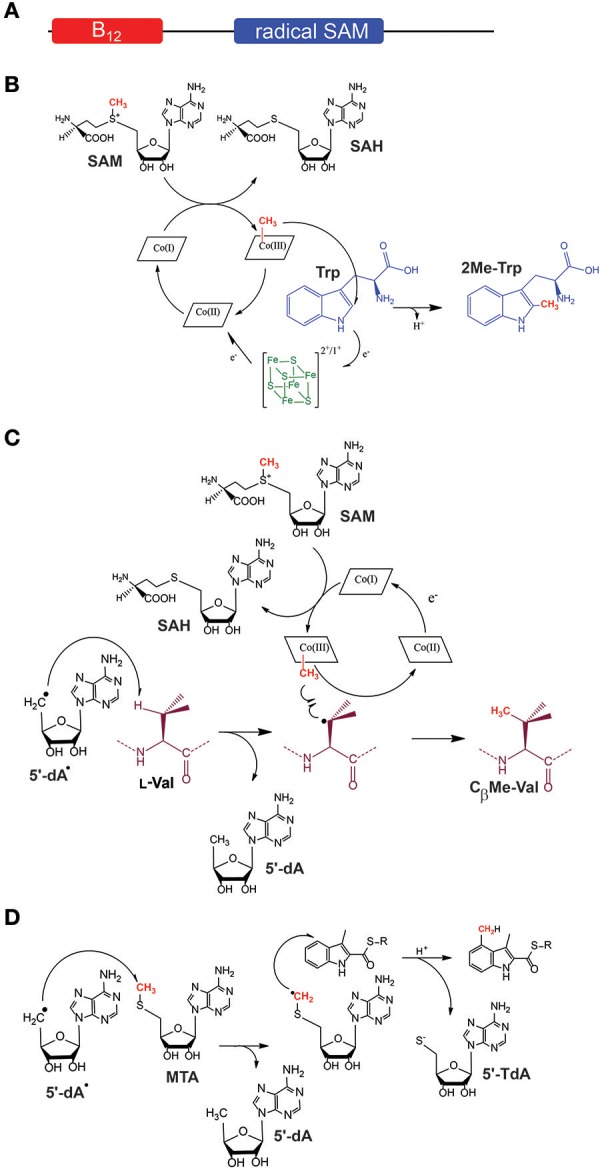
Proposed mechanisms for class B (B_12_-dependent) and class C radical SAM enzymes. **(A)** General architecture of B_12_-dependent radical SAM enzymes. **(B)** Proposed mechanism for TsrM, an *sp*2-hybridized carbon methyltransferase. **(C)** Proposed mechanism for PoyC, an *sp*3-hybridized carbon methyltransferase. In contrast to TsrM, PoyC catalyzes SAM homolytic cleavage likely to abstract a substrate C_β_ H-atom. This intermediate is likely to react with a methyl radical species leading to the formation of C_β_ methyl-valine. In both mechanisms, Cob(II)alamin is likely formed. After further reduction, Cob(I)alamin can react with SAM to regenerate methyl-cobalamin for the next catalytic cycle. **(D)** Proposed mechanism for NosN, a class C methyltransferase. NosN uses two molecules of SAM to generate 5′-dA radical and 5′-methylthioadenosine (MTA). The 5′-dA radical abstracts an H-atom from the methyl group of MTA leading to the addition of the radical intermediate to the C-4 of the indolyl substrate. After C-S bond cleavage, 5′-thioadenosine (5′-TdA) and the methylated indole product are released.

According to its protein sequence, TsrM contains a radical SAM domain between residues 247–447 with the canonical radical SAM motif: C^253^xxxC^257^xxC^260^ and a cobalamin (B_12_)-binding domain located in its *N*-terminus region between the residues: 9-151 (Figure 3). In the presence of SAM and cobalamin, it was possible to reconstitute *in vitro* the activity of this enzyme and to demonstrate that TsrM catalyzes methyl transfer to the C2 of tryptophan (Pierre et al., 2012). By using SAM specifically deuterated on the methyl group, it was revealed for the first time that B_12_-dependent radical SAM enzymes transfer a methyl group from SAM to cobalamin before a final transfer to the substrate. In addition, this study showed that all three H-atoms of the SAM methyl group were transferred from SAM to 2-Me Trp (Pierre et al., 2012). Altogether, these results rationalized the net retention of the configuration monitored in earlier feeding experiments (Frenzel et al., 1990) and confirmed a double methyl displacement during catalysis.

Unexpectedly, contrary to all other radical SAM enzymes, the activity of TsrM proved to be independent of the presence of an external electron donor (Pierre et al., 2012). Furthermore, it was shown that TsrM does not reductively cleave SAM into 5′-dA (Figure 1) but uses SAM only as a source of methyl groups with the concomitant production of *S*-adenosyl L-homocysteine (SAH). Although surprising, these results were consistent with the fact that TsrM transfers a methyl group on an *sp*2-hybridized carbon atom which cannot be activated by radical H-atom abstraction. Further study showed that TsrM is a promiscuous enzyme able to transfer methyl on various indole derivatives (Benjdia et al., 2015). By exploiting its substrate promiscuity, it was established that beside the H-atom on the C2, no other H-atoms are involved in catalysis (Benjdia et al., 2015). These studies rigorously demonstrated that TsrM is the first radical SAM enzyme which does not catalyze the reductive cleavage of SAM, although requiring an [4Fe-4S] cluster and the CxxxCxxC motif for activity (Pierre et al., 2012; Benjdia et al., 2015).

Another unusual feature of TsrM was the fact that the enzyme can use either methylcobalamin (MeCbl) or hydroxycobalamin (OHCbl) for catalysis (Benjdia et al., 2015). This result was in sharp contrast with other B_12_-dependent methyltransferases such as methionine synthase (Banerjee and Matthews, 1990; Chen et al., 1995) or the corrinoid iron-sulfur protein (Kung et al., 2012). Indeed, under anaerobic and reducing conditions, OHCbl is readily converted into cob(II)alamin which is a well-known inactive state of these enzymes which catalyze S_N_2 methyl transfer reactions.

Two mechanisms can account for the transfer of a methyl group to tryptophan (Lewis et al., 2010). One scenario implies the transfer of a methyl radical from MeCbl to tryptophan. Such radical species was unprecedented within an enzyme active site until recently. However, an elegant study on methyl-coenzyme M reductase provided evidences that enzymes can produce such highly reactive intermediate (Wongnate et al., 2016). In addition, it has been shown that synthetic catalysts exclusively transfer methyl radical on the C2 of tryptophan (Gui et al., 2014). After methyl transfer to tryptophan, reduction of cob(II)alamin into cob(I)alamin would allow a facile nucleophilic attack on SAM, regenerating MeCbl for the next catalytic cycle (Figure 3).

The other scenario implies an S_N_2-type mechanism. This mechanism requires the formation of the challenging 4,7-dihydro intermediate, in order to selectively transfer a methyl group on the C2 position (Bartoli et al., 2010) and does not propose a clear function for the radical SAM cluster, while recent investigations have shown that the unique [4Fe-4S] cluster of TsrM binds SAM (Blaszczyk et al., 2016) and is critical for the enzyme activity (Benjdia et al., 2015). However, a recent study published by Booker and collaborators has provided experimental evidences in favor of this mechanistic alternative (Blaszczyk et al., 2017). These observations warrant further studies to elucidate the mechanism of this intriguing enzyme.

While TsrM does not directly transfer a methyl group on the RiPP backbone, several B_12_-dependent radical SAM enzymes (Ding et al., 2016b; Zhou et al., 2016) have been proposed to catalyze such post-translational modifications. In the biosynthetic pathway of bottromycin (Figure 2), three methyltransferases are responsible for the transfer of a methyl groups on three distinct amino acid residues (Val, Phe, and Pro) (Huo et al., 2012). In the biosynthetic pathway of polytheonamide A, two methyltransferases (PoyB and PoyC) have been identified as the likely candidates responsible for methyl transfer on Met, Gln, Ile, Thr, Val and the formation of a *t*-butyl group. Interestingly, in both biosynthetic operons, the methyltransferases were proposed to transfer methyl groups on C_β_-atoms (Figure 2) and to possess an unconventional Cx_7_Cx_2_C motif, suggesting a putative common origin (Parent et al., 2016).

Recently, it was demonstrated that PoyC is an authentic radical SAM enzyme catalyzing methyl transfer to a synthetic peptide designed based on the sequence of polytheonamide A (Parent et al., 2016). In the presence of SAM and MeCbl, it was shown that PoyC catalyzes methyl transfer to a valine residue. However, in sharp contrast to TsrM, PoyC produces 5′-dA during catalysis along with SAH. This feature is shared by other B_12_-dependent radical SAM enzymes catalyzing C(*sp*3)-methylation (Kim et al., 2013; Marous et al., 2015), suggesting they abstract a substrate H-atom (Figure 3). It hence appears that, depending of the carbon-atom to be substituted (*sp2* vs. *sp3*), B_12_-dependent radical SAM enzymes have evolved distinct mechanisms.

### B_12_-independent radical SAM enzymes: NosN & TbtI

More recently, the involvement of another class of radical SAM enzymes in methyl transfer reactions has been established. These enzymes, which are characterized by sequence homologies with HemN (Layer et al., 2002) are referred as class C radical SAM methyl-transferases in contrast to B_12_-dependent radical SAM enzymes which belong to class B methyl-transferases (Zhang et al., 2011b). Two enzymes, TbtI and NosN, involved in, respectively, the biosynthesis of the thiopeptide thiomuracin (Mahanta et al., 2017) and nosiheptide (Ding et al., 2017a,b), have been recently characterized. In an unexpected manner, it has been proposed that NosN uses the SAM cofactor to generate not only 5′-dA but also 5′-methylthioadenosine (MTA) (Ding et al., 2017a). Labeling studies have shown that MTA is the methyl donor leading to indole C4 methylation (Figure 3). The identification of a shunt product resulting from the addition of MTA to the substrate supports this conclusion (Ding et al., 2017b). NosN thus appears to use an unprecedented mechanism for the methylation of *sp2*-hybridized carbon-atoms (Figure 3). It remains to determine if other class C methyltransferases such as TbtI use a similar mechanism.

## Radical SAM enzymes catalyzing thioether bond formation in RiPPs

Another major class of radical SAM enzymes recently investigated is the one catalyzing the formation of unusual C_α_-thioether bonds, characteristic of the RiPP family of sactipeptides (Figure 2). Thioether bonds are a well-known post-translational modifications commonly found in natural products. They are characteristic of the broad and biologically important family of lanthipeptides (Arnison et al., 2013; Hetrick and van der Donk, 2017). In lanthipeptides, the so-called lanthionine bridges are formed by the Michael addition of a cysteine sulphydryl group to a dehydrated serine or threonine residues. These thioether bonds are thus formed between the C_β_-atom of a serine or threonine residue and the *S*-atom of a cysteine residue (Tang et al., 2015).

**Figure 4:**
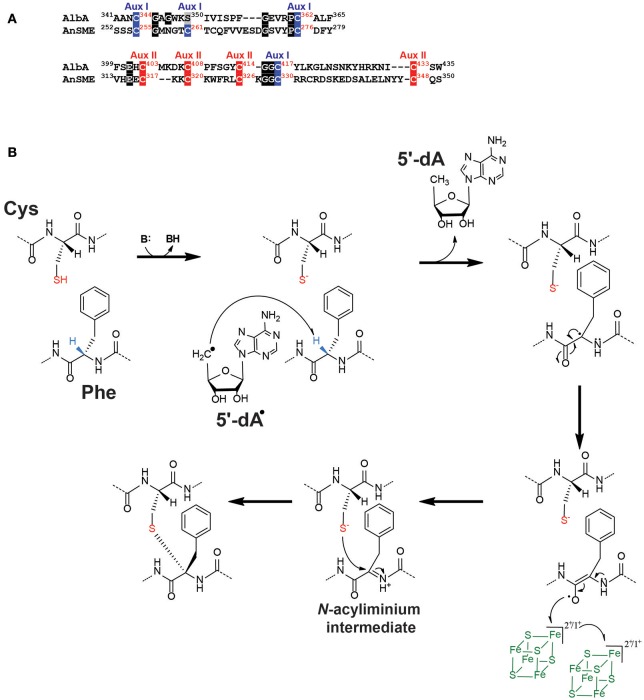
Thioether bond formation by radical SAM enzymes. **(A)** Sequence alignment of AlbA and anSME showing the cysteine residues involved in the coordination of the [4Fe-4S] clusters present in the SPASM-domain. Cysteine residues involved in the auxiliary cluster I (Aux I) and auxiliary cluster II (Aux II) are indicated in blue and red, respectively. Numbers indicate the respective positions of the cysteines in the protein sequences of AlbA and anSME. **(B)** Proposed mechanism for AlbA. AlbA catalyzes H-atom abstraction on C_α_-atom. The carbon-centered radical rearranges leading to the formation of an *N*-acyliminium intermediate. This intermediate is quenched by the thiolate group of a cysteine residue resulting in the formation of a C_α_-thioether bond. The auxiliary clusters I and II are proposed to serve as an electron conduit.

In contrast to lanthipeptides, the formation of thioether bonds in sactipeptides has remained elusive until recently. The first sactipeptide isolated is subtilosin A produced by *Bacillus subtilis*. It was identified 30 years ago and characterized as a cyclic peptide containing cross linkages involving cysteinyl residues (Babasaki et al., 1985). Its structural analysis revealed that it possesses unconventional thioether bonds (Kawulka et al., 2004) involving the C_α_-atoms of distinct amino acids (i.e., threonine and phenylalanine). Interestingly, the subtilosin A biosynthetic operon was shown to encode for a radical SAM enzyme: AlbA, which was presumed to be responsible for the formation of these unique thioether bonds (Zheng et al., 2000). Based on its protein sequence, AlbA appeared to belong to an emerging class of radical SAM enzymes (Benjdia et al., 2010; Grell et al., 2015; Benjdia and Berteau, 2016) called SPASM-domain radical SAM enzymes (Haft and Basu, 2011). The founding member of this class is the so-called anaerobic Sulfatase-Maturating enzyme (anSME) (Berteau et al., 2006; Benjdia et al., 2007a,b, 2008, 2009; Grove et al., 2008) which catalyzes the post-translational modification of a critical active-site cysteine or serine residue into a C_α_-formylglycine (Benjdia et al., 2007b, 2008), a key catalytic residue of sulfatases (Benjdia and Berteau, 2016). The biochemical (Benjdia et al., 2007b, 2008), mutagenesis (Benjdia et al., 2010) and structural (Goldman et al., 2013) investigations of anSME revealed that it coordinates two additional [4Fe-4S] clusters in its *C*-terminal SPASM-domain. These two [4Fe-4S] clusters are coordinated by 8 cysteine residues with Cys^255^, Cys^261^, Cys^276^ and the remote Cys^330^ coordinating the most buried [4Fe-4S] cluster (also called “*auxiliary I*”) and Cys^317^, Cys^320^, Cys^326^, and Cys^348^ coordinating a surface exposed [4Fe-4S] cluster (also called “*auxiliary II*”) (Figure 4A) (Benjdia et al., 2010; Goldman et al., 2013). Although these two [4Fe-4S] clusters have been shown to be critical for the enzyme activity and most likely involved in an electron transfer pathway (Benjdia et al., 2010), their function is still ill-understood.

Biochemical and *in vivo* studies confirmed that AlbA is a SPASM-domain radical SAM enzyme catalyzing the formation of subtilosin A thioether bonds (Flühe et al., 2012; Himes et al., 2016). However, AlbA possesses only seven conserved cysteine residues in its SPASM domain out of the eight present in AnSME (Figure 4A). It was hence suggested that AlbA contains only one auxiliary [4Fe-4S] cluster proposed to be involved in the coordination of the enzyme substrate. A recent study showed that the seven cysteine residues are all critical for the catalytic activity of AlbA (Benjdia et al., 2016). It is thus more than likely that, similarly to anSME, AlbA coordinates two auxiliary [4Fe-4S] clusters (Benjdia et al., 2016). Interestingly, contrary to anSME which catalyzes C_β_ H-atom abstraction (Benjdia et al., 2009), AlbA has been shown to abstract a C_α_ H-atom (Benjdia et al., 2016) to initiate its reaction. Based on these data, a novel mechanism has been recently proposed for the radical formation of thioether bridges in sactipeptides (Figure 4B). After H-atom abstraction and rearrangement, the formation of a critical *N*-acyliminium intermediate has been postulated. This intermediate would easily trap the nucleophilic thiolate of the cysteine residue and lead to the formation of a C_α_-thioether bond (Figure 4B). In this scenario, the two auxiliary [4Fe-4S] clusters present in the SPASM domain would play the role of an electron conduit like in anSME. The formation of a carbon-sulfur bond by a radical SAM enzyme has been recently investigated in great details by using HydE and a thiazolidine, as an artificial substrate (Rohac et al., 2016). Remarkably, HydE catalyzed the radical addition of a carbon-centered radical directly on the thiazolidine sulfur atom without the assistance of other cofactors such as iron-sulfur clusters.

Since the discovery of subtilosin A, several peptides containing C_α_-thioether bridges have been uncovered including the sporulation killing factor (SkfA) (Liu et al., 2010), thurincin H (Sit et al., 2011) and thuricin CD (Rea et al., 2010). SkfA contains a single thioether bond between a cysteine residue and the C_α_-atom of a methionine residue (Liu et al., 2010). Thuricin CD is a two-component bacteriocin characterized by three thioether bridges involving serine, threonine, alanine or tyrosine residues (Rea et al., 2010). Thurincin H is so far unique by containing four thioether bridges involving asparagine, threonine and serine residues (Sit et al., 2011). The radical SAM enzymes involved in the biosynthesis of these sactipeptides most likely proceed through the same radical-based mechanism which implies the generation of C_α_ centered radical peptide intermediate, as shown for AlbA (Benjdia et al., 2016) and more recently for SkfB (Fluhe et al., 2013; Bruender and Bandarian, 2016a). It is also worth to notice that all the enzymes predicted to catalyze the formation of thioether bonds, contain at least one auxiliary [4Fe-4S] cluster. However, the exact function and number of these clusters is still a matter of debate. Interestingly, thioether bridges formation by radical SAM enzymes has also been reported in bacterial proteins (Datta et al., 2001; Nakai et al., 2015). It was shown that one enzyme, QhpD, catalyzes the formation of C_β_- and C_γ_-thioether bonds between a cysteine residue and a Glu or an Asp residue, respectively, in quinohemoprotein amine dehydrogenase. The fact that the same enzyme activates always the carbon atom adjacent to the carboxylic function (i.e., the C_β_- or the C_γ_-position in Glu and Asp, respectively) might have mechanistic implications which remain to be deciphered.

## Radical SAM enzymes catalyzing epimerization reactions

With the discovery of the polytheonamide A biosynthetic pathway, it appeared that radical SAM enzymes could also catalyze peptide epimerization (Freeman et al., 2012). Peptide epimerization has been sporadically reported in bio-active peptides extracted from eukaryotes including mollusks, arachnids and mammals (Bansal et al., 2008). These epimerizations occur at the penultimate end of peptides and the enzymes catalyzing these modifications use a reversible mechanism. Because of the apparent reversibility of the reaction, these enzymes have been called peptide isomerases, although we have still a limited knowledge of them (Bansal et al., 2008).

Contrary to eukaryotes, in polytheonamides, epimerization occurs within the peptide-backbone with an almost perfect 1,3-epimerization pattern (Figure 2). Recently, other epimerized peptides related to polytheonamides have been identified and called proteusins. These peptides contain from two (Morinaka et al., 2014) to eighteen epimerizations (Freeman et al., 2012), located on various amino acid residues. It has been shown *in vivo* that radical SAM enzymes are responsible for these epimerizations and that a solvent derived H-atom is introduced in the D-residues (Morinaka et al., 2014).

**Figure 5:**
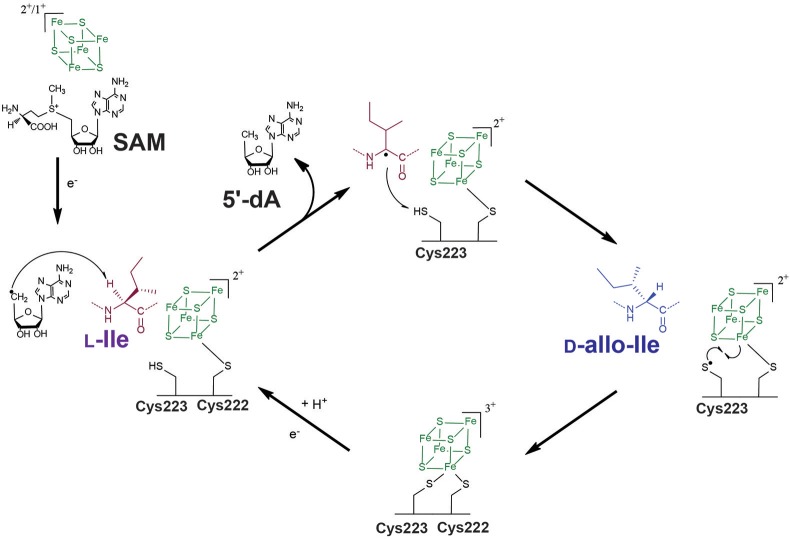
Proposed mechanism for YydG, a radical SAM epimerase. Similarly to AlbA (Figure 4B), YydG catalyzes C_α_ H-atom abstraction leading to the loss of the amino acid stereochemistry. An enzyme cysteine residue (Cys^223^) provides an H-atom to the carbon-centered radical intermediate, resulting in peptide epimerization. The additional [4Fe-4S] cluster located in the *C*-terminus end of the protein, likely assists the quenching of the thiyl radical formed during the reaction and regenerates the cysteine H-atom donor, for the next catalytic cycle.

Until recently, it was believed that these enzymes were restricted to few bacterial taxa including cyanobacteria (Morinaka et al., 2014) and tectomicrobia (Wilson et al., 2014). However, very recently, a novel radical SAM epimerase called YydG was discovered in *Bacillus subtilis* (Benjdia et al., 2017b). This enzyme belongs to a biosynthetic operon which induces LiaRS, a major component of the bacterial cell envelope stress response (Butcher et al., 2007). *In vitro* study of this radical SAM enzyme has revealed that it catalyzes epimerization of two hydrophobic amino acid residues (i.e., Ile and Val) (Figure 5). Similarly to AlbA (Benjdia et al., 2016), YydG was shown to reductively cleave SAM and catalyze C_α_ H-atom abstraction (Benjdia et al., 2017b). However, in a fascinating manner, once the C_α_-centered radical is formed on the substrate peptide and the stereochemistry of the amino acid residue is lost, the radical intermediate is not quenched by a cysteine from the substrate (Benjdia et al., 2016) but it reacts with a cysteine residue from the enzyme (Benjdia et al., 2017b). Thus, in contrast to AlbA which catalyzes thioether bond formation, YydG introduces amino acid epimerization (Figure 5).

Cysteine residues have been shown to serve as H-atom donor in several radical SAM enzymes. The most thoroughly investigated enzyme is spore photoproduct lyase (SPL) (Chandor et al., 2006; Benjdia, 2012; Benjdia et al., 2012, 2014; Berteau and Benjdia, 2017), a radical SAM enzyme catalyzing DNA repair (Berteau and Benjdia, 2017). It has been demonstrated that SPL requires a critical cysteine residue to terminate the reaction (Chandor-Proust et al., 2008; Benjdia, 2012; Benjdia et al., 2012, 2014). In its absence, side-products are formed resulting from the quenching of the substrate radical-intermediate by components of the reaction medium (Chandor-Proust et al., 2008; Benjdia et al., 2012). Interestingly, the enzyme activity can be rescued by placing a cysteine residue within the enzyme active-site, in the vicinity of the substrate (Benjdia et al., 2014). Other enzymes, such as PolH involved in peptidylnucleoside biosynthesis (Lilla and Yokoyama, 2016) and NeoN catalyzing aminoglycoside epimerization (Kudo et al., 2014), have also been shown to require a cysteine residue as H-atom donor. Cysteine residues appear thus to fulfill this function in many radical SAM enzymes and, although the mechanism of the epimerases involved in polytheonamide and proteusins biosynthesis has not been characterized, these enzymes are likely to use a similar strategy for catalysis.

As demonstrated by mutational and spectroscopic studies, YydG likely contains, in addition to the radical SAM cluster, an [4Fe-4S] cluster in its *C*-terminal region similarly to SPASM-domain radical SAM enzymes. It has been proposed that this cluster quenches the thiyl radical formed on the protein, leading to the recycling of the cysteine H-atom donor (Benjdia et al., 2017b) (Figure 5). Finally, it was shown that the epimerized peptide produced by YydG and called epipeptide, efficiently inhibits bacterial growth (Benjdia et al., 2017b). While its biological function has yet to be deciphered, bioinformatic analysis revealed that these epipeptides are present within major species of the human microbiota (Benjdia and Berteau, 2016; Benjdia et al., 2017b). Epipeptides are thus novel members of the RiPP family and likely contribute to the homeostasis of this complex ecosystem (Benjdia et al., 2011).

## Novel strategies for C-C bond formation in RiPPs

**Figure 6:**
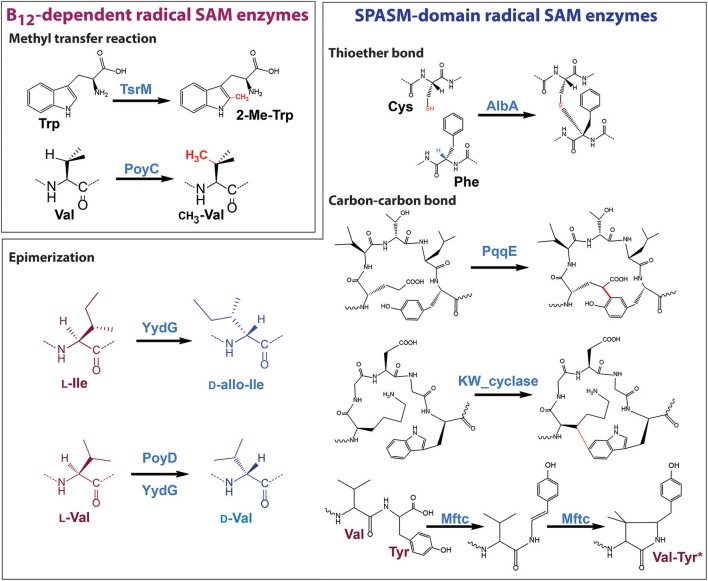
Post-translational modifications catalyzed by Radical SAM enzymes on RiPPs.

In the last years, several radical SAM enzymes have been shown to be involved in the formation of C-C bonds leading to the cross linking of amino acids residues in RiPPs. Two enzymes, PqqE, involved in pyrroloquinoline quinoline biosynthesis (Wecksler et al., 2009) and the KW_cyclase from *Streptococcus thermophilus* (Schramma et al., 2015; Benjdia et al., 2017a), catalyzing the formation of cyclic peptide called “streptide,” have been demonstrated to catalyze the formation of C-C bond between an amino acid side chain (Glu or Lys) and an aromatic residue (Tyr or Trp), respectively (Figure 6). These two enzymes appear to be mechanistically related to radical SAM enzymes catalyzing the formation of thioether bonds. Indeed, their mechanism likely involves the generation of a carbon-centered radical (on Cγ and C_β_, respectively) and they possess a *C*-terminal SPASM-domain likely containing two auxiliary [4Fe-4S] clusters.

In a fascinating manner, a novel enzyme called MftC enzyme has been shown to use a different mechanism to form C-C bond in RiPPs. Indeed, while PqqE and the K_W cyclase likely catalyze the radical addition of a carbon-centered radical on an aromatic residue, MftC catalyzes the oxidative decarboxylation of tyrosine (Bruender and Bandarian, 2016b; Khaliullin et al., 2016) followed by a second H-atom abstraction on a valine residue (Khaliullin et al., 2017), to form a unique cross-linked Val-Tyr^*^ (Figure 6).

## Complex rearrangements in RiPPs

One prominent example of enzyme catalyzing complex rearrangements in RiPPs is NosL which converts L-tryptophan into 3-methyl-2-indolic acid during the biosynthesis of nosiheptide (Zhang et al., 2011a). This enzyme has been shown, contrary to all other radical SAM enzymes, to catalyze H-atom abstraction on the tryptophan amino hydrogen atom (Nicolet et al., 2014; Sicoli et al., 2016). After H-atom abstraction and carboxyl fragment migration, 3-methylindolic acid is formed. Interestingly, NosL has been shown to be a highly promiscuous enzyme able to modify a broad range of tryptophan analogs (Bhandari et al., 2015, 2016; Ji et al., 2015, 2016; Ding et al., 2016a; Qianzhu et al., 2016) allowing a detailed mechanistic study of this unique enzyme.

To conclude, the study of radical SAM enzymes catalyzing RiPP post-translational modifications has shown that these enzymes use common chemical strategies to have access to a wide range of transformations (Figure 6). For instance, radical SAM enzymes catalyzing epimerization, thioether and C-C bond formations all appear to produce carbon-centered radicals (on C_α_, C_β_, or C_γ_). These radical intermediates can react either with a cysteine residue from the protein (YydG) or the substrate (Subtilosin A) leading to the formation of epimerization (Benjdia et al., 2017b) and thioether bonds (Benjdia et al., 2016), respectively. In the case of PqqE and the KW_cyclase, the carbon-centered radical reacts with an aromatic residue leading to the formation of a C-C bond while MftC requires a second SAM cleavage to complete its reaction. In addition, all these radical SAM enzymes contain auxiliary [4Fe-4S] clusters in their *C*-terminal domain which appear to be critical for the enzyme catalysis (Benjdia et al., 2010, 2016, 2017b,a). Interestingly, mutagenesis studies performed on AlbA (Benjdia et al., 2016) and the K_W cyclase (Benjdia et al., 2017b) have shown that these enzymes likely contained three [4Fe-4S] clusters with two of them in their SPASM domain. These clusters have been predicted to be coordinated by at least seven cysteine residues with five cysteine residues highly conserved within a Cx_2−4_Cx_5_Cx_2−3_Cx_14−18_C motif and two remote cysteine residues (Benjdia et al., 2017a). Very recent structural analyses of radical SAM enzymes catalyzing thioether or carbon-carbon bond formation have demonstrated these predictions to be correct (Davis et al., 2017; Grove et al., 2017) but shown a plasticity in the coordination of the two iron-sulfur clusters present in the SPASM-domain. It is more than likely that other variations will be identified within the SPASM-domain in the coming years.

In contrast, B_12_-dependent radical SAM enzymes catalyzing RiPP methyl transfer reactions, despite sharing similar domains and cofactor requirements, appear to use different chemistries in order to methylate *sp*2 and *sp*3-hybridized carbon-atoms (Figure 6).

Questions such as the role of the leader peptide or of accessory proteins remain to be solved. Indeed, enzymatic systems such as MftC (Bruender and Bandarian, 2016b; Khaliullin et al., 2016) and PqqE (Barr et al., 2016) require an accessory protein for activity while other radical SAM enzymes have been shown to be dependent of the presence of a leader peptide (Schramma et al., 2015; Benjdia et al., 2017a) or a protein domain called RiPP precursor peptide recognition element (i.e., RRE or PqqD-like domain) (Burkhart et al., 2015). However, many radical SAM enzymes catalyze their transformations in the absence of these elements (Benjdia et al., 2016, 2017b). The understanding of the molecular basis of the interactions between radical SAM enzymes and RiPPs is still at its early stage. It will require thorough structural and biochemical investigations of these novel enzymatic systems to draw a comprehensive picture of these emerging enzymes.

Similarly, future investigations of novel biosynthetic pathways notably within the human microbiome, are likely to uncover novel transformations catalyzed by radical SAM enzymes and to further expand the repertoire of RiPPs, as recently exemplified by the discovery of epipeptides (Benjdia et al., 2017b). These novel RiPPs are a promising source of bioactive molecules and antibiotics with useful clinical applications. Finally, the extraordinary potential of radical SAM enzymes to introduce chemically challenging modifications will also undoubtedly lead to innovative approaches in synthetic biology and RiPP engineering.

## Author contributions

AB and OB wrote the manuscript. All authors made intellectual contribution and approved the manuscript for publication.

### Conflict of interest statement

The authors declare that the research was conducted in the absence of any commercial or financial relationships that could be construed as a potential conflict of interest.
